# Effect of different decellularization protocols on reendothelialization with human cells for a perfused renal bioscaffold of the rat

**DOI:** 10.1186/s12896-022-00767-1

**Published:** 2023-03-16

**Authors:** Johannes Sauter, Hannes Degenhardt, Jutta Tuebel, Peter Foehr, Philipp Knoeckel, Kira Florian, Fiona Charitou, Rainer Burgkart, Andreas Schmitt

**Affiliations:** 1 Department of Orthopedics and Sports Orthopedics, Klinikum Rechts der Isar der Technischen Universität München, Munich, Germany; 2 Department of Medicine II, LMU Klinikum München, Munich, Germany; 3 Division of Sports Orthopedics, Klinikum Rechts der Isar der Technischen Universität München, Munich, Germany; 4 Krankenhaus St. Adolf-Stift, Reinbek, Germany; 5 Orthopädisches Fachzentrum Weilheim, Weilheim, Germany

**Keywords:** Scaffold, Kidney, Decellularization, Recellularization, Endothelium, SDS, Tissue engineering, Artificial

## Abstract

**Background:**

Scaffolds for tissue engineering can be received by whole organ decellularization while maintaining the site-specific extracellular matrix and the vascular tree. One among other decellularization techniques is the perfusion-based method using specific agents e.g. SDS for the elimination of cellular components. While SDS can disrupt the composition of the extracellular matrix and impair the adherence and growth of site-specific cells there are indications that xenogeneic cell types may benefit from protein denaturation by using higher detergent concentrations. The aim of this work is to investigate the effect of two different SDS-concentrations (i.e. 0.66% and 3%) on the ability of human endothelial cells to adhere and proliferate in an acellular rat kidney scaffold.

**Material and methods:**

Acellular rat kidney scaffold was obtained by perfusion-based decellularization through the renal artery using a standardized protocol including SDS at concentrations of 0.66% or 3%. Subsequently cell seeding was performed with human immortalized endothelial cells EA.hy 926 via the renal artery. Recellularized kidneys were harvested after five days of pressure-controlled dynamic culture followed sectioning, histochemical and immunohistochemical staining as well as semiquantitative analysis.

**Results:**

Efficacy of decellularization was verified by absence of cellular components as well as preservation of ultrastructure and adhesive proteins of the extracellular matrix. In semiquantitative analysis of recellularization, cell count after five days of dynamic culture more than doubled when using the gentle decellularization protocol with a concentration of SDS at 0.66% compared to 3%. Detectable cells maintained their endothelial phenotype and presented proliferative behavior while only a negligible fraction underwent apoptosis.

**Conclusion:**

Recellularization of acellular kidney scaffold with endothelial cells EA.hy 926 seeded through the renal artery benefits from gentle decellularization procedure. Because of that, decellularization with a SDS concentration at 0.66% should be preferred in further studies and coculture experiments.

## Introduction

Tissue engineering is a research branch of regenerative medicine that is focusing on creating artificial tissues or organs for experimental research or clinical aspects such as transplantation. Cells, scaffolds and biological stimuli are three obligatory components for its application collectively called the triad of tissue engineering [1]. While all of them being equally important, scaffolds represent the basic structure of tissue engineered constructs giving their quality enormous significance for successful implementation. Therefore, a primary goal is to obtain scaffolds as similar as possible to the physiologic extracellular matrix (= ECM) providing the same environmental conditions in vitro as in vivo. Based on that some key scaffold requirements are bioactivity, biocompatibility and degradability [2]. Another essential aspect in scaffold generation is to provide sufficient nutrient supply for reseeded cells. Regarding the diffusion distance of oxygen which is approximately 100–200 μm it must be ensured that there is at least one capillary vessel located every 400 μm [3]. Otherwise, cellular metabolism cannot be maintained leading into apoptosis and tissue destruction.

Besides many existing techniques and biomaterials for artificial scaffold fabrication, whole organ decellularization provides a further method that follows a different approach. It aims for lysis and complete removal of cells and their remnants thus the native ECM with its vascular tree remains. Therefore, the perfusion-based method using detergents such as sodium dodecyl sulfate (= SDS) or triton x-100 is commonly applied [4]. In 2008, the first whole organ decellularization was accomplished using the example of a rat heart [5] and afterwards expanded on rat liver [6], lung [7] and kidney [8]. While absence of nuclear cell components is a primary criteria for efficacy [9] leaving the ECM as unharmed as possible is at least equally important. As decellularization agents tend to damage the ECM it is of elemental importance to finely balance their concentration and exposure time. Corresponding to the site-specific acellular proportion of all tissues, the ECM is produced by resident cells like fibroblasts, endothelium or epithelium and undergoes permanent change depending on current requirements [10, 11]. It fulfills various elemental function for tissue morphogenesis hence being much more than just a passive bystander. By defining stiffness, collagens and elastin as the main structure proteins affect stem cell differentiation and therefore provide biomechanical functions [12]. Furthermore, reticular collagen IV represents an essential adhesion protein for endothelial cells [13]. Laminin, fibronectin, and tenascin are important examples for glycoproteins mainly based in the vascular basement membrane [14, 15]. As ligands for ECM-receptors such as integrins and syndecans they play a vital role in cell attachment, adhesion, and migration [16, 17]. With growth factors like Vascular Endothelial growth factor (= VEGF), Fibroblast growth factor (= FGF), or Epidermal growth factor (= EGF) bound to proteoglycans, the ECM is furthermore able to influence cell behavior and differentiation [18]. Regarding all these native properties and functions of the ECM it is comprehensible to use decellularized scaffolds in tissue engineering although reseeding with endothelial cells is needed. This so-called reendothelialization remains a significant hurdle of tissue engineering and could not yet be overcome applying either the cell-based or scaffold-based method [19]. While decellularized tissues provide an organ-specific vascular tree that fulfills anatomical requirements such as well-ordered capillaries, homogenous reseeding of endothelial cells has not yet been accomplished. Consecutively, thrombogenesis in tissue engineered constructs remains a major limiting factor for its successful application.

Optimized decellularization protocols are required to achieve complete antigen removal from tissues while minimizing side effects on the ECM. With the intention to exploit acellular kidney scaffolds for bone tissue engineering our group discovered that decellularization with SDS at concentrations at 0.5%, 0.66%, 1% and 3% did not significantly change the immunogenicity of the kidney scaffold for xenogeneic cell types [20]. We additionally proved that human umbilical vein endothelial cells (= HUVECs) and human osteoblasts could be seeded through the renal artery leading to adhesion, proliferation and bone-specific remodeling of the ECM [21]. For xenogeneic (i.e. human) but not for allogenic (i.e. rat) osteoblasts we furthermore observed improved recellularization of acellular renal scaffold by performing aggressive decellularization at 3% SDS compared to 0.66%. In detail, when decellularization was performed at 3% SDS, human osteoblasts showed improved proliferation and cellular viability in acellular rat kidney scaffolds after 14 days of dynamic culture. These findings suggest that xenogeneic cell types may rather benefit from intense and therefore denaturing decellularization protocols.

The aim of the work is to investigate the ability of endothelial cells to attach and proliferate in acellular kidney scaffold depending on the aggressiveness of the beforehand used decellularization protocol. With that it should be evaluated whether the findings of our group regarding improved recellularization with xenogeneic osteoblasts after denaturing decellularization at 3% SDS could also be transferred on human endothelial cells. In order to avoid known disadvantages of primary endothelial cells such as HUVES, the well characterized human immortalized endothelial cell line EA.hy 926 [22] was used for the present study. This includes their limited life spawn and tendency to senesce as well as donor-depended properties leading to impaired comparability of results [23].

## Results

### Characterization of cells

**Fig. 1:**
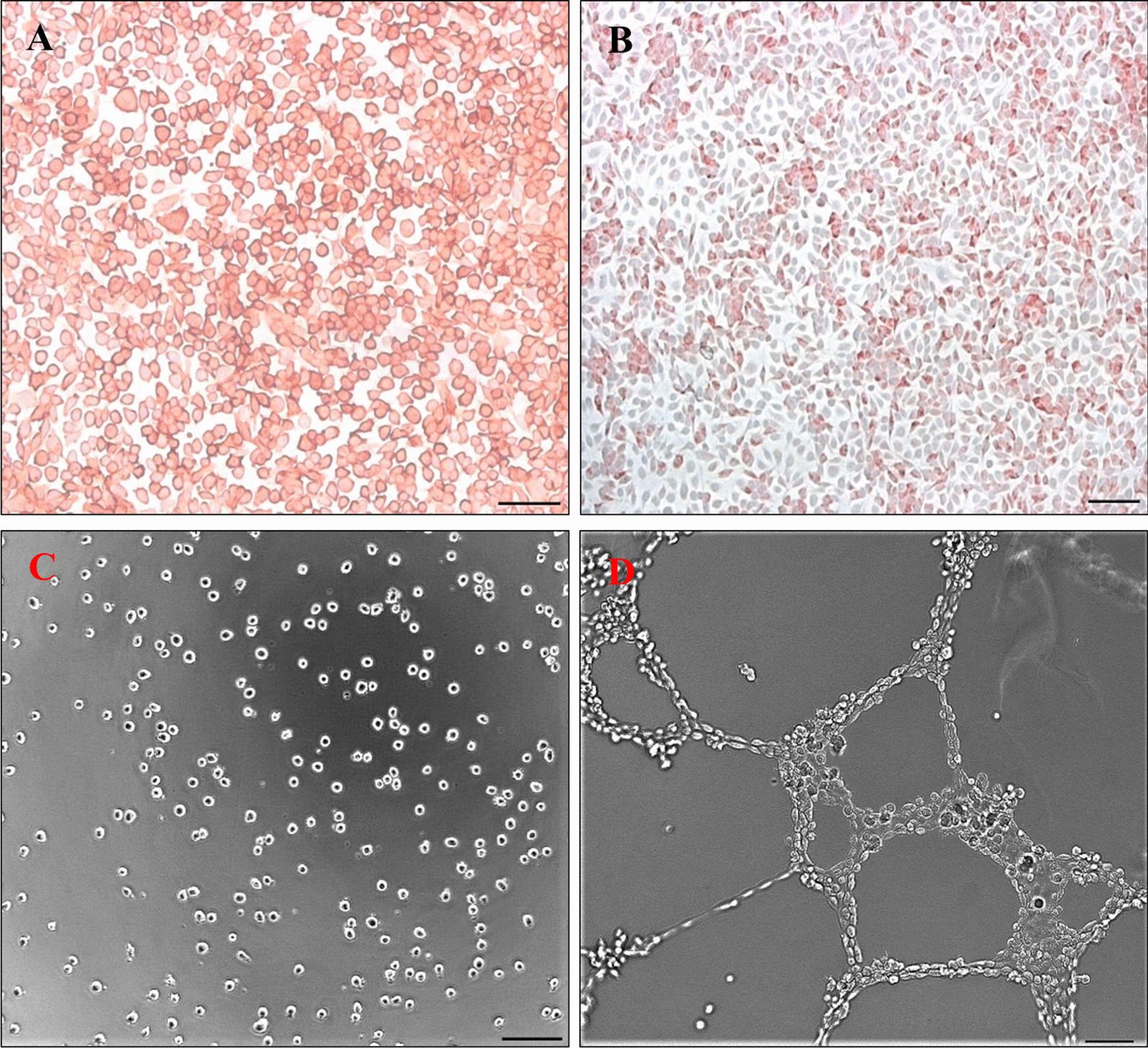
Characterization of human endothelial cells EA.hy 926. Immunocytochemical staining (A + B) and in-vitro-angiogenesis assay (C + D). Cells showed a strong expression of CD-31 (**A**) and vWF (**B**). After being seeded on extracellular matrix solution (**C**) they formed a fine network of tubules within 18 h (**D**). Scale bar represents 100 μm

To confirm endothelial phenotype endothelial cells EA.hy 926 were stained for CD-31 (Fig. 1A) and von-Willebrand-factor (Fig. 1B) after preceded cultivation on chamber slides. Cells presented strong expression for these antigens while isotype control remained negative. Additionally, in-vitro-angiogenesis-assay (abcam® catalog number: ab204726) was performed to evaluate tubule formation. Initially, seeded endothelial cells appeared round to oval (Fig. 1C). After 18 h of culture on extracellular matrix solution, confluent cells with longitudinal cytoplasmatic branches could be observed forming a reticular network of vessel like structures (Fig. 1D). Negative control without use of extracellular matrix solution did not result in change of cell formation. Representative images are visualized in Fig. 1.

### Decellularization

**Fig. 2:**
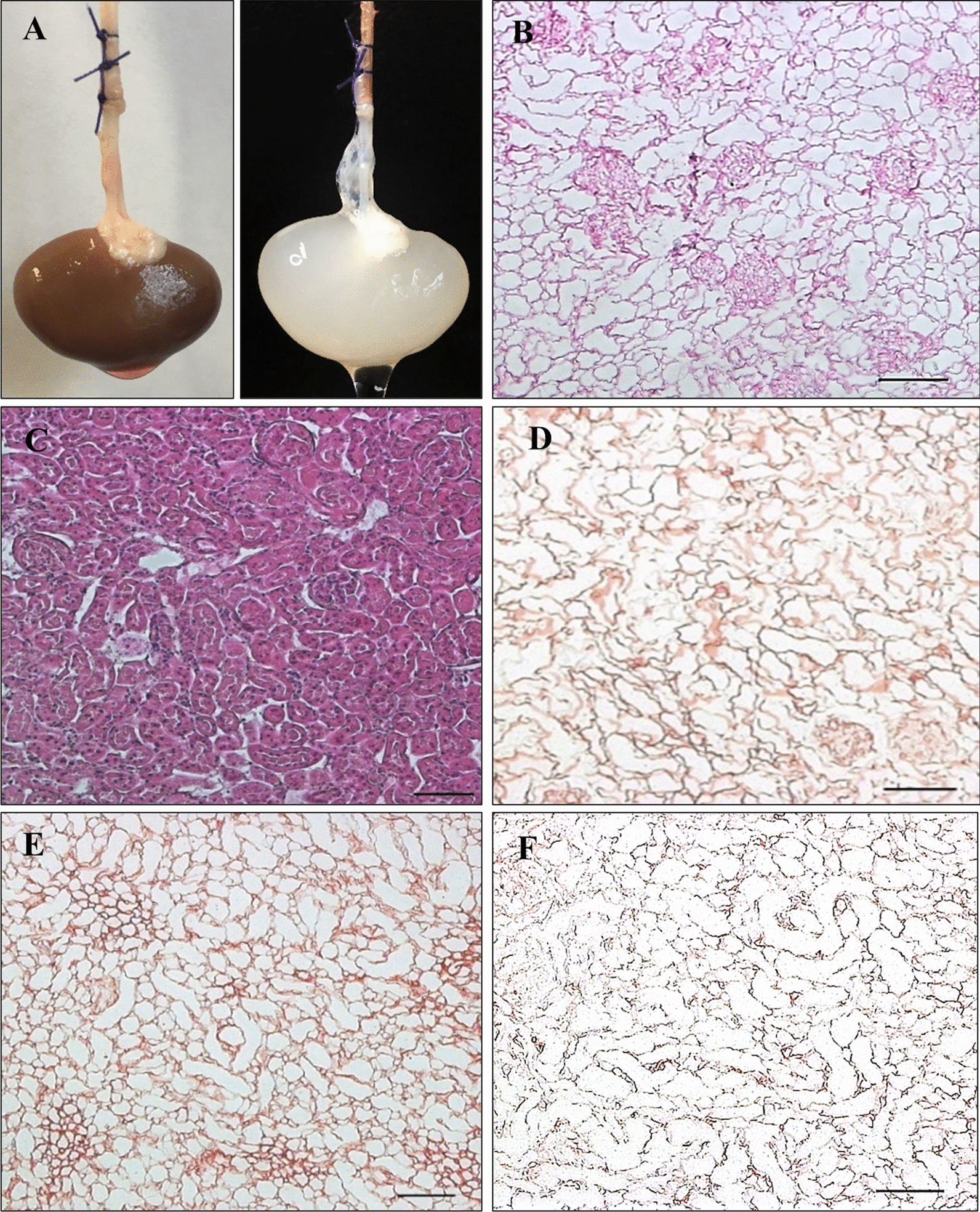
Efficacy of the decellularization procedure. Native Rat kidney before (**A**, left) and after (**A**, right) decellularization at 0.66% SDS. After the decellularization procedure, the rat kidney scaffold appeared milky to translucent. Microscopic images of kidney parenchyma before (**B**) and after (**C**, **D**–**F**) decellularization at 0.66% SDS with preservation of microstructure and absence of cellular components in H.&E.-staining (**C**). Preservation of Laminin (**D**), Fibronectin (**E**) and Collagen IV (**F**). Scale bar represents 100 μm

Whole organ decellularization of rat kidneys was accomplished by constant pressure-controlled perfusion with SDS at a concentration of either 0.66% or 3%. After completion of this procedure the macroscopic appearance of the parenchyma was milky to translucent, and the native dark red color was completely washed out. Histologically, the parenchyma remained intact and well organized with preservation of physiological structure and visible glomeruli, renal tubuli and vascular basement membranes. Furthermore, there was no detection of cellular components or remnants. In immunohistochemical staining the presence of laminin, fibronectin and collagen IV was verified. Representative images are illustrated in Fig. 2.

**Fig. 3:**
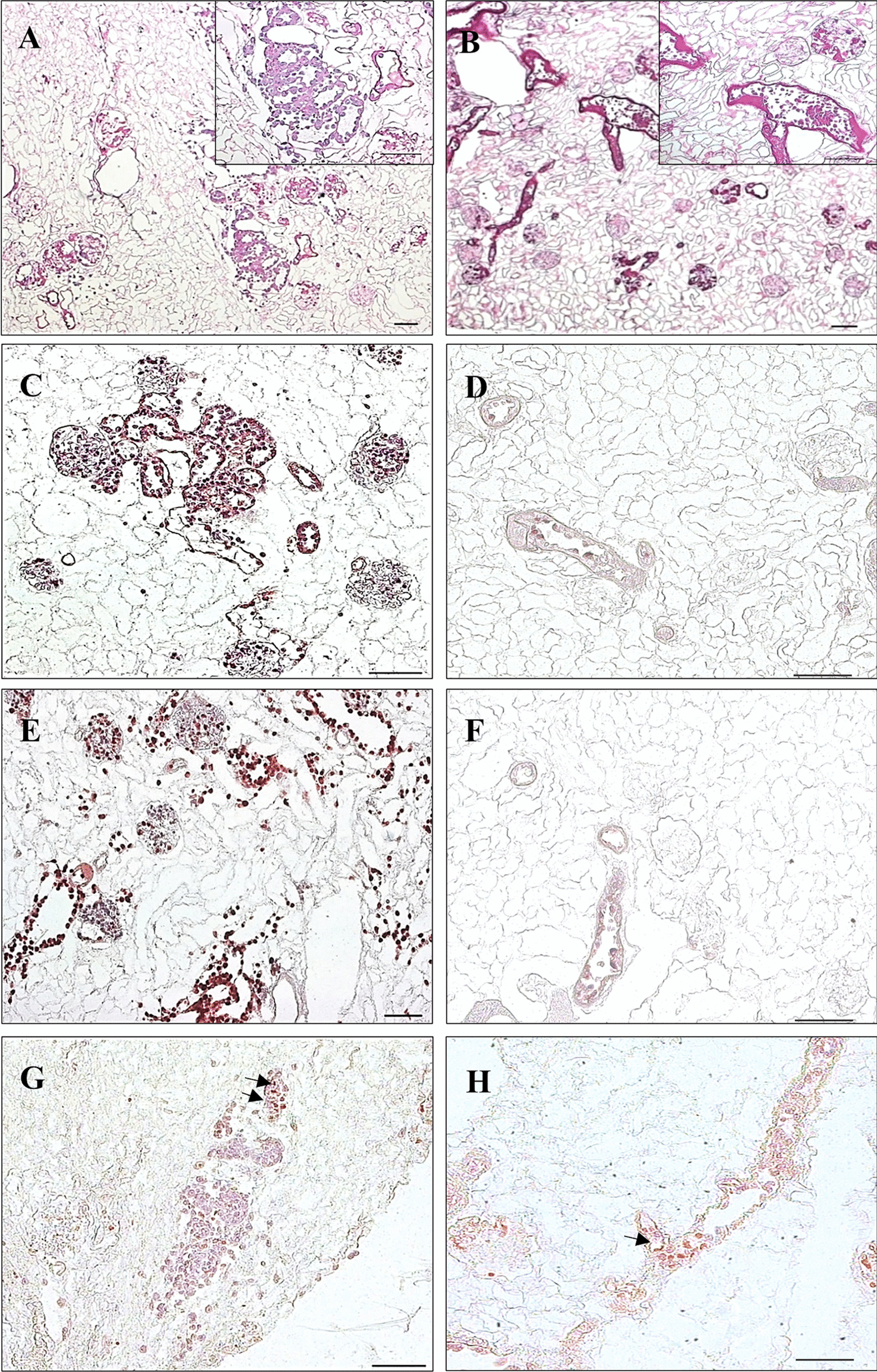
Representative microscopic images after 5 days of rat kidney scaffold recellularization with human endothelial cells EA.hy 926. The left column displays multiple attached endothelial cells covering the vascular basement membrane as a monolayer after 0.66% SDS while after 3% SDS there was only a small number of detectable cells (right column). Evaluation of recellularization success was primarily obtained with HE-staining (**A**, **B**). Staining for CD-31 (**C**, **D**) confirms preservation of endothelial phenotype and staining for PCNA (**E**, **F**) indicates proliferative behavior. Apoptotic cells were detected by ISNT (**G**, **H**, arrows). Scale bar represents 100 μm. N = 3 for each column

RecellularizationFor recellularization of acellular kidney scaffolds endothelial cells EA.hy 926 were seeded through the renal artery and cultivated for 5 days at dynamic conditions according to the above descripted techniques. Microscopically, recellularization success depended on aggressiveness of the beforehand used decellularization protocol. Representative images of 3 kidneys for each SDS concentration are displayed in Fig. 3.

After being performed with 3% SDS, only a small number of cells attached to the vascular basement membrane could be observed. Because of poor cell density, the kidney scaffold seemed almost vacant in all anatomical areas equally. After decellularization with 0.66% SDS however noticeably more cells could be detected covering the vascular basement membrane and the glomeruli partly in a monolayer like manner with a predominant distribution in the cortex area. In immunohistochemical staining cells presented strong positivity for CD-31 and PCNA as confirmation for preservation of endothelial phenotype and proliferative behavior. Only a negligible portion of cells underwent apoptosis as detected with ISNT.

**Fig. 4:**
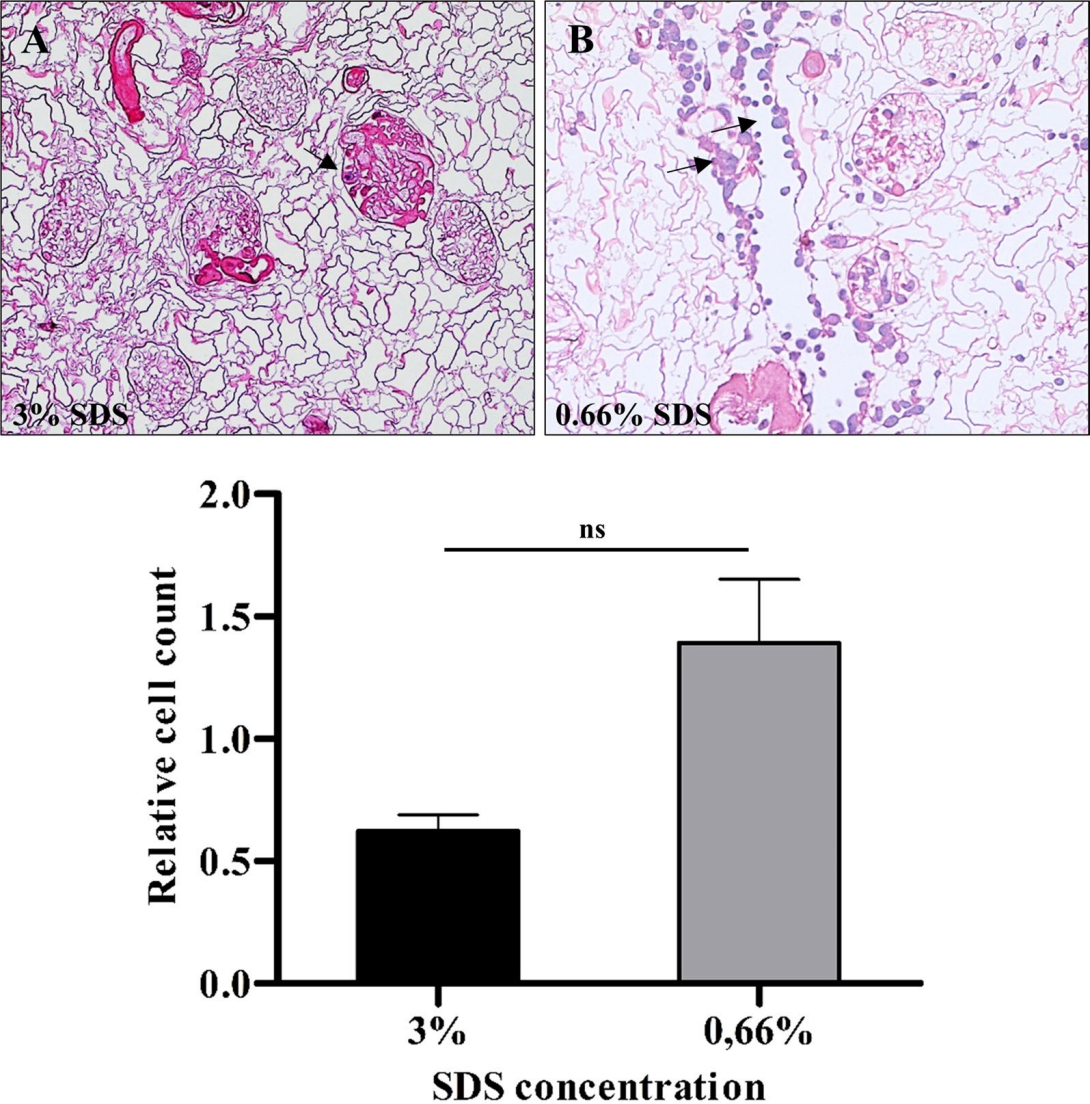
Semiquantitative analysis of relative cell count after 5 days of dynamic culture with endothelial cells EA.hy 926. For cell counting, 20 microscopic images in 200 times magnification from 4 different section levels of each kidney were used. Representative images are shown in **A** for 3%SDS and **B** for 0.66% SDS, respectively. Arrows indicate adherent cells. For improved comparison of recellularization procedures, the relative quantity of adherent cells was then calculated by setting the counted cells in relation to 1 × 10^6^ inserted cells. The mean (± SD) of the relative cell count was 0.62 (± 0.07) and 1.39 (± 0.26) after decellularization at 3% SDS and 0.66% SDS, respectively (*p* = 0.1, two-tailed Mann–Whitney U test). Relative cell count could be more than doubled after using the gentle decellularization protocol at 0.66% SDS (4C). n = 3 for each SDS concentration

For comparison of recellularization success depending on the beforehand used SDS concentration attached cells were counted as described above, representative images are visualized for decellularization at 3% SDS (Fig. 4A) and 0.66% SDS (Fig. 4B) With being set in relation to the number of injected cells relative quantity (i.e., counted cells per 1 × 10^6^ inserted cells) was calculated (Fig. 4C). After decellularization at 3% SDS a mean (± SD) of 0.62 (± 0.07) cells were found per microscopical image. By using SDS at a concentration of 0.66% the relative number of cells more than doubled to a mean (± SD) of 1.39 (± 0.26). The difference of relative cell quantity was statistically not significant (*p* = 0,1; two-tailed Mann–Whitney U test).For both test regimes 3 kidneys were used each.

## Discussion

In the present study we demonstrate that quality of recellularization with endothelial cell line EA.hy 926 in acellular kidney scaffold depends on the insensitivity of the beforehand used decellularization protocol. Specifically, even if statistically not significant, relative cell count after five days of dynamic culture more than doubled after gentle (i.e. 0.66% SDS) compared to aggressive (i.e. 3% SDS) decellularization. Thus, our present results demonstrate that gentle decellularization indicates to offer more suitable culture conditions for the human endothelial cell line EA.hy 926 which benefits attachment and proliferative behavior in acellular renal scaffolds. For culture of human osteoblasts, our group surprisingly discovered improved niche conditions in acellular kidney scaffold after decellularization with SDS at a concentration of 3% compared to 0.66%. These results could not be verified for the human endothelial cell line EA.hy 926. Regarding observations by Burgkart, Tron [21] and Schmitt, Csiki [20] who could already prove suitable growth conditions for human osteoblasts in renal scaffold after decellularization at 0.66% SDS this decellularization protocol should also be preferably used for future coculture experiments. SDS is known for effective removal of cellular components [24] and is more potent to do so even in cell dense tissues compared to other detergents such as triton x-100 [25, 26]. For the purpose of whole organ decellularization it is typically used at concentrations between 0.1 and 1% depending on organ size [27] ranging up to 4% in combination with triton x-100 at a concentration of 3% performed in rat kidneys [28, 29]. At the same time SDS also has adverse effects on the remaining extracellular matrix such as reducing amount of growth factors and sulfated glycosaminoglycans [30, 31] as well as disrupting its ultrastructure [32]. Besides concentration of the used agents, their exposure time is the second key variable defining how significant these effects are. Compared to decellularization protocols of rat kidney described in the literature [28, 29, 33–35], the method by Burgkart, Tron [21] used in this study is not only time saving but also comparatively gentle due to short exposure time. Efficacy was furthermore confirmed by absence of cellular remnants, intact ultrastructure and positive immunohistochemical staining for laminin, fibronectin, and collagen IV. However, aggressive decellularization with SDS at 3% led to insufficient recellularization with only singular cells adherent to the vascular basement membrane. As compared to noticeably improved results after gentle decellularization at 0.66% SDS this might most probably be caused by an impairment of endothelial cell niche conditions in the acellular kidney scaffold due to SDS-induced alterations of the ECM.[31, 32]. Observations of our group although indicated improved results for long-term cultivation of human osteoblasts seeded via the ureter after denaturing decellularization with SDS at 3% compared to 0.66%. Above mentioned alterations on the ECM might therefor result in an appropriate niche even for xenogeneic cells types. It is known that perfusion pressure and shear stress differ in between the tubules and the vasculature system leading to uneven physical effects on cellular binding for both compartments. In detail, the physiologic perfusion pressure in the renal artery of a rat measures over 100 mmHg [36] and remains almost 60 mmHg in the glomerular capillaries [37] while it is only approximately 8.3 mmHg in the bowman´s capsule [38]. In which compartment cells are reseeded might therefore have enormous implications for recellularization success due to their physical properties as cells being seeded in the vasculature have to resist and overcome higher shear stress which can disturb adherence and proliferation [39]. For recellularization of acellular kidney scaffolds, 20–40 × 10^6^ immortalized human endothelial cells EA.hy 926 were injected into the renal artery followed by dynamic culture at perfusion pressure up to 100 mmHg matching physiologic conditions in vivo [36]. As demonstrated in previous studies by Burgkart, Tron [21] reseeding with HUVECs could be successfully performed after decellularization at 0.66% SDS following this procedure. While both mentioned cell types share similar phenotypes [40], expression of integrin β1, -α2 und α5-subunits is significantly lower in endothelial cells EA.hy 926 than in HUVECs impairing their ability to establish a firm adhesion to fibronectin and collagen I in static culture [41]. As perfusion pressure and concomitant shear stress is known to negatively affect reendothelialization [39] weak binding to the ECM can furthermore lead to cell detachment and removal under dynamic conditions. Despite impaired adhesive properties of EA.hy 926 endothelial cells, the present work still demonstrates their ability to adhere and proliferate in acellular kidney scaffold after gentle decellularization at 0.66% SDS. Predominantly in the cortex area, they reached an acceptable density partly forming a monolayer on the vascular basement membrane. With these results endothelial cells EA.hy 926 can be considered as an viable alternative for primary endothelial cells such as HUVECs which were so far frequently used for dynamic culture studies in acellular kidney scaffolds. By applying EA.hy 926 endothelial cells instead of HUVECs, some of their natural limitations can even be overcome. This particularly includes their short life spawn after which they senesce, transform to giant cells, and ultimately undergo apoptosis as well as donor-specific properties leading to less comparable results depending on their origin [23].

A major and yet unsolved problem of reendothelialization is to obtain an even distribution of endothelium throughout the vascular system. The dispersion of cells within the scaffold is strongly affected by how they are brought into it during the seeding process giving this step enormous importance [42]. As performed in the present study, the primary method to insert endothelial cells into acellular kidney scaffolds is seeding through the renal artery. On this way cells will distribute within arterial vessels and glomeruli but only barely reach the peritubular capillaries [43]. To overcome this issue, successive injecting anterograde though the renal artery and retrograde through the renal vein is described to be beneficial [34]. Furthermore applying negative pressure during seeding [33] or preincubation with CD-31 antibodies [44] were reported giving significant improvements. According to existing literature our experiments also resulted in insufficient distribution with a relatively low cell count in the medulla area even after decellularization with SDS at 0.66%. As discussed above, optimal seeding techniques for the endothelial cell type EA.hy 926 need to be further investigated.

However, the present work has some limitations. First, no recellularization of acellular kidney scaffold was performed after decellularization with SDS at concentrations in between 0.66% and 3%. For recellularization purpose only one cell type (i.e. EA.hy 926 endothelial cells) was utilized giving less opportunity for comparative evaluation. To our knowledge comparative analysis do not exist in the literature and must be further investigated. Only a total of 6 experiments with n = 3 for each group (i.e., SDS at 0.66% and 3%) were obtained and analyzed making statistical evaluation rather unsuitable. Each experiment was stopped after 5 days of dynamic culture to evaluate the generale suitability of human endothelial cells EA.hy 926 for attachment and proliferation in acellular kidney rat kidney scaffold. On the other hand, no long-term studies e.g., 14 or 21 days were applied. Lastly, no coating or preincubation of the vascular system within the acellular renal scaffold for potential optimization of recellularization was tested.

## Conclusion

In the present study we could demonstrate that gentle decellularization of rat kidney with a concentration of SDS at 0.66% improves subsequent reseeding of acellular kidney scaffold with human immortalized endothelial cells EA.hy 926 in comparison to aggressive decellularization at 3% SDS. Therefore, decellularization protocols with SDS at a concentration of 0.66% should be preferably used for further endothelial cell studies.

## Material and methods

### Cell culture

Human endothelial cells EA.hy 926 (ATCC® CRL-2992™) were obtained in frozen state and cultivated in Dulbecco’s Modified Eagle’s Medium (DMEM) ATCC® 30–2002™ with 10% fetal calf serum and Primocin™ (Invivogen) at a final concentration of 1%. Cell splitting und subculture were performed using the trypan blue assay and a cell strainer with a mesh size of 40 μm to reduce cell clotting. Media was changed within that process or at least every 3 days. Before experimental use, the endothelial phenotype was verified by Cluster of Differentiation 31 (= CD-31, Dako) and von-Willebrand-factor (= vWF, Dako) staining. Furthermore, an in-vitro-angiogenesis-assay was performed for confirmation following provider instructions (abcam® catalog number: ab204726). Therefore, endothelial cells were seeded on extracellular matrix solution and incubated for no longer than 18 h. For experiments endothelial cells were used in the 3rd–7th passage.

### Preparation and decellularization of rat kidneys

After ethics approval cadavers of Wistar laboratory rats were obtained from the University Hospital of the Technical University of Munich following controlled euthanasia. For this purpose, the laboratory rats received anesthesia following intracardial injection of phenobarbital. Kidneys were dissected and frozen at − 80 °C. For each experiment, the kidneys were thawed, the surrounding soft tissue was removed and an 20G catheter was fixed in the renal artery with surgical knots. Only one kidney was used for each experiment. Subsequently the cannula was adapted to a unit of a decellularization system which was manufactured in our laboratory. With evacuated tubes it was then connected to a pressure-controlled arthroscopic pump (Arthrex Continous Wave II AR-6450). Decellularization was started and performed at ambient temperature at a perfusion pressure of 100 mmHg. The duration of the whole procedure was 200 min including 60 min of SDS in concentration of either 0.66% or 3% and decontamination with penicillin/streptomycin to obtain a sterile scaffold. All steps are illustrated in Table 1.

**Table 1:** Decellularization procedure

Step	Perfusion solution	Duration (min)
1	Destilled water	10
2	SDS*	30
3	Destilled water	10
4	SDS*	30
5	Destilled water	60
6	Pen/Strep	60
Total		200

*At either 0.66% or 3%

### Recellularization

Parenchyma was washed with 5 mL pure media and cells were seeded into the vascular system through the cannula fixed to the renal artery. Therefore 20–40 × 10^6^ endothelial cells EA.hy 926 were suspended in 2 mL culture media and injected manually with a constant flow of 1 mL/min. For each decellularization procedure (i.e. 0.66% or 3% SDS) 3 acellular rat kidneys were recellularized. Decellularized kidneys with SDS at 3% were each reseeded with 20 × 10^6^ endothelial cells. After decellularization at 0.66% SDS, reseeding was performed 20 × 10^6^ endothelial cells in 1 kidney and 40 × 10^6^ endothelial cells in 2 kidneys to evaluate changes in recellularization efficacy. The same procedure was repeated twice with suspension exiting through the renal vein. Kidneys were connected to a sterile mini incubator, drained in media and transferred into a cell culture incubator (37 °C, 5% CO2). Perfusion was started after 5-8 h at 50 mmHg pressure. After 24 h of dynamic culture perfusion pressure was increased to 100 mmHg. A total of 250 mL media was recirculated and changed every 3 days. Dynamic culture was maintained for 5 days. In total 6 Kidneys (n = 6) were used with n = 3 for decellularization at 0.66% SDS and n = 3 for decellularization at 3% SDS.

### Histology

For histologic investigation samples were formalin-fixed and embedded in paraffin. Subsequently they were cut into slices of 4 μm, deparaffinized and rehydrated. Sections were stained with hematoxylin and eosin (= HE). After heat induced epitope retrieval immunohistochemistry was performed using the ABC-method (Vector Laboratories) in combination with Aminoethyl carbazole (= AEC) chromogen (Dako). Primary antibodies (Dako) were used at following concentrations: Anti-CD-31 1:50, anti-vWF 1:50, Proliferating-Cell-Nuclear-Antigen (= PCNA) 1:500, anti-Laminin 1:500, anti-Fibronectin 1:500, anti-Collagen IV 1:25. Secondary antibodies (Vector Laboratories) were applied at a dilution of 1:200, isotype anti-IgG were used as negative controls in corresponding concentration to the primary antibody. Counterstaining was accomplished by hematoxylin. For detection of apoptosis in-situ-nick-translation (= ISNT) was performed according to Gold, Schmied [45].

### Semiquantitative evaluation and statistics

For semiquantitative examination cell counting was performed. Therefore, a total of 20 microscopic images in 200 times magnification from 4 different section levels of each kidney were used. Only cells adherent to the vascular basement membrane were included. In relation to the number of cells injected during seeding process relative cell count was calculated in order to compare experiments with different amounts of seeded cells. Afterwards average and standard deviation of 3 kidneys for each SDS concentration were calculated. For statistics and graphs, GraphPad Prism version 8.3.0 for Windows, GraphPad Software, La Jolla California USA, www.graphpad.com" was used.

## Data Availability

All data generated or analysed during this study are included in this published article.

## Abbreviations

AEC: Aminoethyl carbazole
CD: Cluster of differentiation
ECM: Extracellular matrix
EGF: Epidermal growth factor
FGF: Fibroblast growth factor
H&E: Hematoxylin and eosin
HUVECs: Human umbilical vein endothelial cells
ISNT: In-situ-nick-translation
PCNA: Proliferating-cell-nuclear-antigen
Pen/Strep: Penicillin and streptomycin
SDS: Sodium dodecyl sulfate
VEGF: Vascular endothelial growth factor
vWF: Von-Willebrand-factor
